# The Transmission of Warts

**Published:** 1907-07

**Authors:** 


### THE TRANSMISSION OF WARTS.

Recent experiments show that the popular belief that warts are communicable is warranted. Inoculation of healthy tissue with blood obtained from a wart will cause the growth of a similar excrescence. It is thought that these growths contain an ultramicroscopic germ to which is due the transmission from one person to another. If an emulsion of wart tissue be filtered through a Berkfield filter and injected into healthy tissue, small excrescences appear in the course of several months, which rapidly develop into typical warts. Repeated trials to obtain a culture of the micro-organism, if such really exist, failed. These investigations would favor a soluble toxin instead of a micro-organism. This might easily be deposited upon the towel and thence to another person. At all events, persons who have warts should take measures to have them removed at once.—Dietetic and Hygienic Gazette, June, 1907.

